# Enteral resuscitation with oral rehydration solution to reduce acute kidney injury in burn victims: Evidence from a porcine model

**DOI:** 10.1371/journal.pone.0195615

**Published:** 2018-05-02

**Authors:** Belinda I. Gómez, Matthew K. McIntyre, Jennifer M. Gurney, Kevin K. Chung, Leopoldo C. Cancio, Michael A. Dubick, David M. Burmeister

**Affiliations:** 1 United States Army Institute of Surgical Research, Fort Sam Houston, TX, United States of America; 2 Brooke Army Medical Center, Fort Sam Houston, TX, United States of America; 3 Uniformed Services University of the Health Sciences, Bethesda, MD, United States of America; The University of Manchester, UNITED KINGDOM

## Abstract

Intravenous (IV) resuscitation of burn patients has greatly improved outcomes and become a cornerstone of modern burn care. However, the heavy fluids and vascular access required may not be feasible in austere environments, mass casualty, or delayed transport scenarios. Enteral resuscitation has been proposed for these situations; we sought to examine the effectiveness of this strategy on improving burn-induced kidney injury. Anesthetized Yorkshire swine sustaining 40% TBSA full-thickness contact burns were randomized to three groups (n = 6/group): fluid deprivation, ad libitum water access, or 70 mL/kg/d Oral Rehydration Salt solution (ORS). Urine and blood were collected at baseline (BL), 6, 12, 24, 32, and 48h post-burn, at which point tissue was harvested and CT angiography performed. Although fluid consumption by ad libitum and ORS groups were matched (132±54mL/kg versus 120±24mL/kg, respectively), ORS intake increased urine output compared with water and no water (47.3±9.0 mL/kg versus 16.1±2.5 mL/kg, and 24.5±1.7 mL/kg respectively). Plasma creatinine peaked 6h following burn (1.67±0.07mg/dL) in all animals, but at 48h was comparable to BL in animals receiving water (1.23±0.06mg/dL) and ORS (1.30±0.09mg/dL), but not fluid deprived animals (1.56±0.05mg/dL) (*P*<0.05). Circulating levels of blood urea nitrogen steadily increased, but also decreased by 48h in animals receiving enteral fluids (*P*<0.05). Water deprivation reduced renal artery diameter (-1.4±0.17mm), whereas resuscitation with water (-0.44±0.14 mm) or ORS maintained it (-0.63±0.20 mm;*P*< 0.02). Circulating cytokines IL-1β, IL-6, IFNγ, and GM-CSF were moderately elevated in the fluid-deprived group. Taken together, the data suggest that enteral resuscitation with ORS rescues kidney function following burn injury. Incorporating enteral fluids may improve outcomes in resource-poor environments and possibly reduce IV fluid requirements to prevent co-morbidities associated with over-resuscitation. Studies into different volumes/types of enteral fluids are warranted. While ORS has saved many lives in cholera-associated dehydration, it should be investigated further for use in burn patients.

## Introduction

Worldwide, one of every ten deaths is a result of trauma which is also the number one cause of mortality among individuals under 40 years of age; burns are the fourth most common type of trauma [1]. In the year 2015, over 180,000 deaths were a result of fire or other hot substances [2]. Severe burn injury elicits a complex physiologic response resulting in diminished plasma volume, hypermetabolism, and a profound inflammatory response which often results in multiple organ dysfunction (MOD). The kidneys are frequently affected in MOD and there is a high incidence of acute kidney injury (AKI) that occurs in burn patients [3, 4]. AKI is also independently associated with increased mortality in thermal injury [5–7]. Associated problems of AKI include retention of blood urea nitrogen (BUN), volume overload, reduced antioxidant status, altered immunologic responses, and mitochondrial damage within the kidney [8].

As a surrogate for kidney function, urine output is the main indicator that guides resuscitation with intravenous (IV) fluids in burn patients. Indeed, the realization that IV resuscitation and maintenance of intravascular volume decreases comorbidities such as AKI and maintains end-organ perfusion has revolutionized burn care and improved patient outcomes. Initial fluid volume infusion rates in burn patients are commonly given in the range of 2–4 ml/kg/%TBSA, representing the range of the modified Brooke (2 ml/kg/%TBSA) and Parkland (4 ml/kg/%TBSA) formulas. However, fluid type and volume administered have yet to be standardized, leading to large variation in resuscitation protocols [9]. While IV fluid resuscitation remains the standard treatment for burn patients, the efficacy of oral rehydration therapy has been proposed for decades [10–12]. Past clinical trials and animal experiments utilized various formulations of simple electrolyte solutions and found them effective for the treatment of burn injury [13]. In disaster or resource-limited situations, enteral fluids may be the only option due to a lack of IV fluids or an inability to gain vascular access.

The Oral Rehydration Salt solution (ORS) according to the World Health Organization (WHO) is a simple formula that contains glucose, sodium chloride, potassium chloride, and trisodium citrate. ORS has been successfully used for decades to save millions of lives in third world countries from dehydration secondary to severe diarrhea in conditions such as cholera [14]. This suggests the feasibility of rapidly mobilizing these simple treatments in the wake of large-scale mass casualty incidents. Additionally, the relative ease of ORS implementation (e.g., drinking, or through a nasogastric tube) may aid in preserving organ function in delayed transport scenarios such as prolonged field care or wilderness medicine. An animal study of 40%TBSA burns in swine demonstrated that roughly 93% of ORS was absorbed, leading to greater urine output than with IV fluids [15]. A clinical study of 20 children with 10–20% TBSA burns found similar levels of urine output comparing enteral and IV fluids [16]. More recently, a randomized clinical trial evaluated enteral resuscitation versus IV fluid in adults with >15% TBSA burns and demonstrated greater urine output on day 3 post-burn in patients receiving enteral fluids [17]. These studies suggest ORS is safe and effective for burn injury, which not only may prove life-saving in the austere environments mentioned above, but also may reduce IV fluid requirements when the patient has reached definitive clinical care.

Despite these studies advocating for the feasibility and efficacy of ORS, the potential for enteral fluids to ameliorate burn-related comorbidities remains largely unstudied. As an initial step to demonstrate the efficacy and feasibility of ORS, we used a large animal model of moderate burn injury to assess the effects of ORS at reducing AKI in a controlled environment. We utilized a 40% TBSA burn conscious swine model. We hypothesized that ORS is reno-protective and is superior to water alone at maintaining kidney function and perfusion.

## Materials and methods

### Animals

The animal protocol was reviewed and approved by the Institutional Animal Care and Use Committee at United States Army Institute of Surgical Research (*Protocol number A16-011*). This study has been conducted in compliance with the Animal Welfare Act, the implementing Animal Welfare Regulations, and the principles of the Guide for the Care and Use of Laboratory Animals. Approval was received prior to research and all efforts were made to minimize animal suffering. A completed ARRIVE guidelines checklist is provided as S1 Fig.

Eighteen pre-pubertal female Yorkshire swine weighing 40.2±2.1 kg, free of parasites and infection, were included in this study. Upon arrival to our Institute, animals had a minimum seven-day acclimation period, during which they were singly housed, with ad libitum access to water, and fed a commercial laboratory porcine formulated pelleted diet. Animals were randomly allocated to one of three treatments following thermal injury: fluid deprived (n = 6), ad libitum water access (n = 6), or 70 mL/kg/d of ORS (ORS; n = 6) for 48h. A gravity-fed spigot was customized using a carboy attached to a lixit via long flexible tubing and attached to a drip bowl on the metabolic cage. Fluid intake was carefully monitored by measuring fluid waste caught in the drip bowl that leaked from the spigot or the animal’s mouth as she drank. For the animals receiving ORS, the amount of waste was replaced with fresh ORS to ensure the animal received the entire volume allotted. During the experimental treatment animals had unlimited access to the dry pelleted diet and special dry cookie treats until fasting before anesthetic events. After euthanasia with 10 mL of Fatal Plus (Vortech Pharmaceuticals, Dearborn, MI) at 48 h, kidneys were harvested and weighed and sections were preserved in 10% neutral buffered formalin until further processing. Additionally, 2–3 grams of the kidney was blotted dry and weighed before and after drying in a 60°C oven for calculating wet-to-dry ratio.

### Thermal injury

Creation of the burn wounds and post-operative animal care were performed as previously described [18]. Briefly, animals were anesthetized with an IM injection of tiletamine-zolazepam (Telazol, 6 mg/kg), intubated, and placed on a ventilator with an initial tidal volume at 10mL/kg, a peak inspiratory pressure of 20 cm H_2_O, and respiratory rate of 8 to 10 breaths/min. The ventilator was adjusted to achieve an end-tidal PCO_2_ of 40±5 mm Hg. Animals were maintained on 1% to 3% isoflurane, balance O_2_ anesthesia. Hair was removed from the dorsum, flanks, and legs using clippers and razors with shaving cream. For the sampling of blood, standard cut-down procedures were used to place left and right external jugular vein catheters that were anchored in place and tunneled subcutaneously to the back of the neck. Large (9x15 cm) and small (5x5 cm) custom-designed brass blocks equipped with a thermocouple were maintained at 100±0.2°C by a temperature controller. Heated probes were placed against the skin for 30 s to produce full-thickness burn injuries as previously described [19]. This procedure was repeated until 40% of the TBSA was burned [20]. Animals did not receive intravenous resuscitation fluids throughout the study.

### Animal care

All animals were given a one-time intramuscular injection of 0.1–0.24 mg/kg Buprenex-HCl Sustained Release (Veterinary Technologies/ZooPharm, Windsor, CO), which provides analgesia for up to 72 hours, immediately prior to the creation of the burn wounds. Burn wounds were covered with Ioban antimicrobial dressings (3M, St. Paul, MN) for the duration of the experiment, which were replaced if wounds were exposed. Animals recovered and were kept in a metabolic cage (dimensions 41’L x 16’ W x 44’ H) for collection of urine and monitoring of their enteral fluid intake. Feed was given once animals were awake and standing independently. Approximately 24 and 48 h following burn injury, animals were sedated with Telazol (6 mg/kg) to collect blood samples and monitor physiological parameters (heart rate, respiratory rate, and rectal temperature). During research period, individual animals were monitored at a minimum hourly (during normal work hours) by the veterinary technician staff, veterinarians, research staff, or husbandry staff for signs of distress. This was routinely done in a hands on fashion daily for all animals (S2 Fig), as well as documented twice a day on pain and distress sheets (S3 Fig) after injury. Additionally, monitoring was continuously done remotely via animal room camera access. If animals showed signs of distress (e.g., vocalization, jumping) they were administered midazolam (0.1–0.25 mg/kg) IM for light sedation. In these scenarios, a sedative over additional analgesia was chosen because the root cause of distress was the animals’ environment (i.e., the metabolic cage) and not pain per se. This was determined due to previous experience with a lack of distress when returned to the home cage [21], and an initial attempt at sham controls exhibiting increased aversion to the metabolic cage.

### Computed tomography (CT) angiography

At baseline and 48 h, renal perfusion, volume, and renal artery diameter were assessed with contrast-enhanced angiography. Animals were anesthetized as described earlier, positioned prone, and 40 mL of contrast agent (Isovue-370; Iopamidol 755 mg/mL; contains sodium 0.053mg and organically bound iodine 370 mg/mL) was injected via ear vein catheter and CT angiographies were performed. Renal artery diameter, kidney volume and perfusion were quantified using Vitrea Advanced Version 6.7.4 (Vital Image Inc., Minnetonka, MN). Both right and left kidneys were selected using 2D slices to reconstruct the entire kidney and the measurement tool gave volume and Hounsfield units of the whole organ. For all parameters measured, changes from baseline to 48 h post burn were calculated for individual animals.

### Histology

Upon euthanasia at 48 h, kidney samples were immediately preserved in 10% neutral buffered formalin, embedded in paraffin wax, and sectioned into 4-μm slices. Periodic Acid Schiff (PAS) staining was performed according to the manufacturer’s instructions (Sigma Life Science, St. Louis, MI) and tubular degeneration was scored on a scale of 0–5 for both distribution (0- none, 1- scattered, 2- <10%, 3-10-25%, 4–25–50%, and 5- >50%) and severity (0- none, 5- severe) by a blinded histopathologist. The scores were added together, leading to a score of 10 being the worst possible. Whole kidney slices were imaged using an AxioScanZ1 slide scanner (Carl Zeiss, Thornwood, NY). Images of entire sections were put through automated quantification of colors with ImageJ software version 1.51d (Bethesda, MD). The PAS color deconvolution tool was used to separate the pink and blue channels. Software-acquired measurements for each channel included the mean intensity and density.

### Blood and urine analysis

Urine samples were collected into 50-mL tubes and blood samples were collected into K_2_ EDTA-containing tubes and centrifuged at 4,300 x g for 10 min, aliquoted, and stored at -80°C until analysis. If there was no urine output overnight, a foley catheter was inserted while the animal was under anesthesia at 24 and 48 h as described above (6mg/kg Telazol IM), and 10mL of urine was aspirated for urinalysis. Superoxide dismutase (SOD) kit was purchased from Cayman (Ann Arbor, MI) and performed according to the manufacturers’ protocol for plasma. Free hemin was quantified in the plasma using a commercially available colorimetric assay kit (BioVision, Milpitas, CA) according to the product inset directions. For cytokine analysis, a porcine-specific multiplex kit (EMD Millipore, Billerica, MA) was used according to the manufacturer’s instructions. Blood samples were also collected into a lithium-heparin-containing tube and centrifuged at 4,300 x g.

Serum and urine biochemical values were analyzed on a Siemens Dimension Xp and Plus Clinical Chemistry System. For complete blood count, blood was collected into K_2_ EDTA containing tubes and analyzed with the Abbott Cell-Dyn 3700 system. For venous blood gas analysis, 1 mL of blood was collected and one drop was loaded into an iSTAT CG4+ cartridge. The cartridge was read using the iSTAT Portable Clinical Analyzer (Abbott Point of Care, Princeton, NJ). Finally, creatinine clearance (i.e., glomerular filtration rate, GFR) was calculated at each timepoint by the following equation: ((Creatinine_urine_ x Volume_urine_)/ (Creatinine_plasma_) x Time_min_). Values across time for each animal were averaged to represent total creatinine clearance.

### Statistical analysis

Statistical analysis was performed using JMP® (SAS institute, Inc, Cary, NC). Data with repeated measures were analyzed using 2-way analysis of variance method (ANOVA) followed by Tukey’s or t-test for multiple comparisons. For analysis of histology, protein, fluid intake, urine output, GFR, kidney volume, artery diameter, and wet-dry ratios a 1-way ANOVA and Tukey’s multiple comparison tests were performed. For these analyses, non-parametric testing was utilized where appropriate (e.g., histological analysis and GFR) or when Brown-Forsythe testing revealed that the variances amongst groups were different. All data are presented as mean ± standard error of the mean (SEM) using GraphPad Prism, which was also used to run linear regression analysis. Significance was set at *P* < 0.05.

## Results

### Burn injury alters physiological parameters

All animals recovered in a metabolic cage. They displayed an appetite the first day of the study but not on the second. No animals died prior to scheduled euthanasia. Seven animals (39%) required midazolam for mild sedation, but maintained full consciousness in the metabolic cage. By h 48 all animals displayed elevated body temperature (BL: 38.21±0.03 vs 48 h: 40.06±0.09°C), respiratory rate (BL: 34±5 vs. 48 h: 43±3 breaths/minute), but not heart rate (BL: 138±3 vs. 48 h: 138±4 beats/minute). Additionally, Table 1 provides physiological parameters demonstrating severity of illness but no significant effect of treatment. Burn injury increased circulating white blood cell count by 6 h which remained elevated throughout the duration of the study (*P* < 0.05 vs baseline). All animals displayed alkalemia by 6 h following burn injury that returned to normal values by 24 h. Venous blood base excess in the extracellular fluid compartment (BEecf) in the fluid-deprived and the ORS group was elevated by 6 h (*P* < 0.05) relative to BL values.

**Table 1:** Burn injury alters WBC count, pH, glucose, and BEecf.

	WBC (1x10^3^/μL)			pH			Glucose (mg/dL)			BEecf (mmol/L)		
Time Point	Fluid deprived	Water	ORS	Fluid deprived	Water	ORS	Fluid deprived	Water	ORS	Fluid deprived	Water	ORS
BL	20.52±1.43	19.03±0.67	16.60±1.18	7.42± 0.02	7.44±0.01	7.42±0.01	66.95±7.84	72.36±9.68	51.01±8.34	5.60±0.51	6.80±0.49	4.17±1.74
6 h	#28.94±2.29	#28.15±2.93	#24.71±2.14	#7.51±0.02	7.47±0.01	ᵻ7.49±0.01	#545.83±282.49	517.61±280.27	293.13±77.35	#10.20±1.83	9.20±1.20	#8.33±1.20
12 h	#27.49±1.86	#26.56±2.61	#26.41±1.23	#7.50±0.03	7.47±0.02	7.46±0.01	211.15±33.01	305.20±80.17	#360.60±68.99	8.20±1.28	9.40±0.98	6.60±0.98
24 h	#25.51±1.88	#24.52±2.16	#25.48±1.67	7.41±0.01	7.44±0.01	7.41±0.03	180.03±28.60	161.23±20.52	159.98±22.60	#11.00±0.89	9.80±1.60	#8.25±1.38
32 h	#33.32±4.21	#32.90±2.92	#31.23±1.61	7.45±0.06	7.43±0.02	7.40±0.03	199.06±41.77	305.55±42.13	172.30±25.17	7.80±2.08	8.40±0.87	4.17±2.07
48 h	#25.74±3.34	#24.25±2.01	#24.71±2.16	7.41±0.03	7.44±0.01	7.41±0.01	257.10±95.20	98.90±4.78	115.81±25.34	9.25±1.60	8.60±1.16	6.67±0.88

White blood cell count, pH, glucose, and BEecf in blood samples were collected at baseline (BL) 6, 12, 24, 32, and 48 h post burn injury. Values are presented as mean ± SEM and a # indicates a significant (*P* < 0.05) difference from the BL value.

### ORS reduces burn-induced acute kidney injury

**Fig 1:**
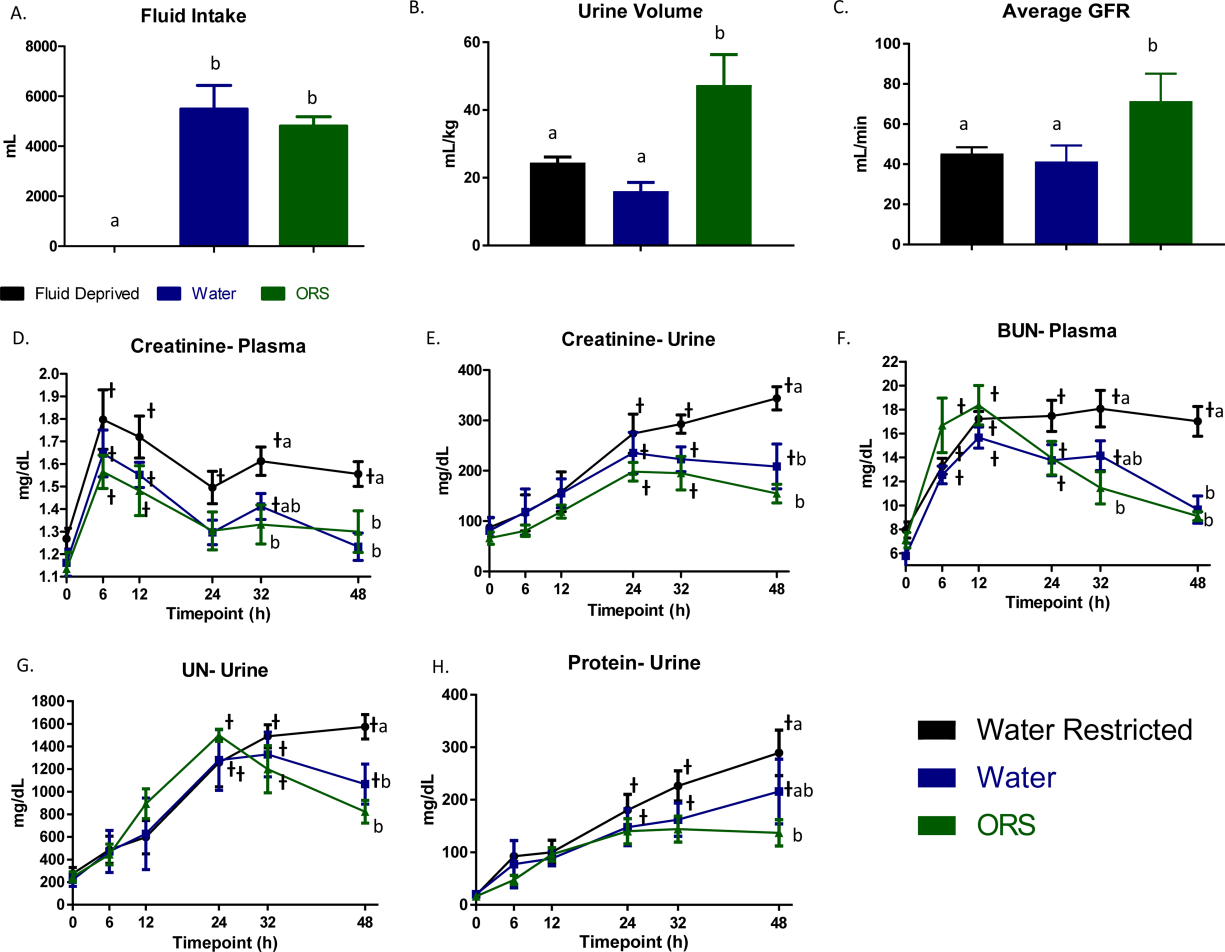
ORS increases urine output and positively alters burn-induced biochemical markers. Total fluid intake (A) urine output volume (B), and (C) Average glomerular filtration rate (GFR) throughout the duration of the study. Levels of creatinine (D, E) and urea nitrogen in the plasma (F) and the urine (G). Urinary protein (H). Means ± SEM with a different superscript are significantly different (*P* < 0.05) between treatments for indicated time point and a ᵻ indicates a significant (*P* < 0.05) difference from the BL value.

The total fluid volume consumed over the 48 h by the ad libitum water and 70 ml/kg/d ORS was not significantly different (5,488 ± 947 mL (132±54 mL/kg) versus 4,812 ± 373 mL (120±24 mL/kg), respectively; Fig 1A). However, ORS consumption led to a significantly greater total urine output, which was nearly tripled when compared with water and doubled when compared with fluid-deprived groups (1,894 ± 361 mL versus 664 ± 113 mL, and 902 ± 47 mL respectively; *P* < 0.05). Additionally, urine output normalized for weight is still greatest with ORS (47.3±9.0 mL/kg) followed by water (16.1±2.5 mL/kg) and finally fluid deprived (24.5±1.7 mL/kg; Fig 1B). Average GFR of the 48 h period was greatest in animals receiving ORS (71.5±13.6 mL/min) when compared to fluid-deprived (45.3±3.4 mL/min) and the water group (41.4±7.9 mL/min).

Plasma creatinine peaked at 6 h in all groups at an average of 1.67±0.07 mg/dL, indicating a moderate level of acute kidney injury by Kidney disease: Improving Global Outcomes stage 2 criteria (Fig 1C). However, the effect of enteral fluids again became apparent, wherein the fluid-deprived group (1.56±0.06 mg/dL) had greater (*P* < 0.05) circulating creatinine when compared with water (1.23±0.06 mg/dL) and ORS (1.30±0.09 mg/dL) groups at 48 h. Urinary creatinine mirrored that of plasma (Fig 1D). A steady increase in urinary creatinine following burn was noted in all animals, but at 48 h the levels began to diverge according to fluid intake. Specifically, both water and ORS groups reached maximum levels (235.3±40.7 and 198.0±18.5 mg/dL; respectively) by 24 h, while fluid deprivation resulted in a further increase of 266.9±45.3 mg/dL. At 48 h, significant differences (*P* < 0.01) in urinary creatinine levels were detected between animals receiving fluids (208.7±44.4 mg/dL 154.8±18.5 mg/dL for water and ORS, respectively) and those that did not (343.8±23.1 mg/dL).

These same temporal shifts are also seen with BUN. Maximal levels of BUN in the plasma were achieved 12 h following burn and also significantly diverged by 48 h (Fig 1E). At that time, plasma BUN in the water and ORS groups returned back to baseline levels (9.65±1.14 and 9.10±0.37 mg/dL respectively) whereas fluid deprivation led to significantly higher levels of circulating BUN (17.01±1.24 mg/dL; *P* < 0.05). Levels of urinary urea nitrogen were significantly greater than baseline by 24 h in all animals; however at 48 h the ORS (822.6±100.2 mg/dL) group was similar to the water (1,067.8±177.2 mg/dL) but significantly lower than the fluid deprived group (1,575.0±109.4 mg/dL; *P* < 0.05; Fig 1F).

Total protein in the urine was elevated by 32 h following burn in fluid deprived and water animals. At 48 h animals receiving ORS had lower levels (*P* < 0.01) of urinary protein when compared with fluid deprived animals (137.1±25.0 mg/dL vs. 289.3±43.4 mg/dL; Fig 1G). The group of animals receiving water (215.7±61.9 mg/dL) was intermediate to the fluid deprived and the ORS groups and therefore not significantly different between either treatment group at 48 h.

## Enteral resuscitation prevents vasoconstriction of the renal artery

**Fig 2:**
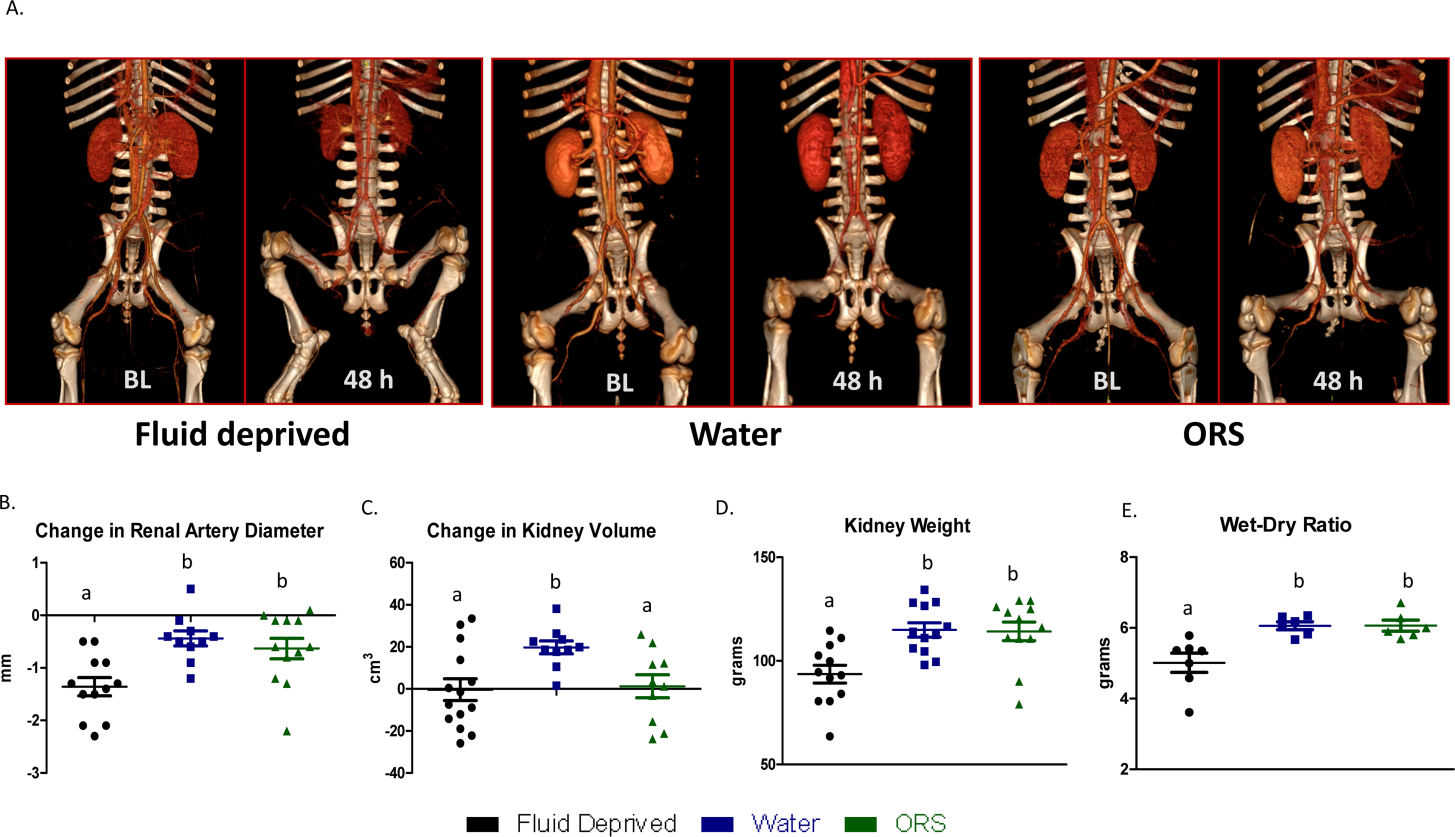
Enteral resuscitation prevents reduction in renal artery diameter. Computed tomography (CT) scanning (A) was performed pre-injury and immediately prior to euthanasia (termination of experiment 48 h). Renal artery diameter (B), kidney volume (C), weight (D), and wet:dry ratios (E) were quantified. For all parameters measured, changes from baseline to 48 h post burn are represented as mean ± SEM. Groups with different superscripts are significantly different (P < 0.05).

To assess renal perfusion and artery diameter, CT angiograms were taken at baseline and 48 h. Representative CT images from animals in each treatment group visually depict changes in the renal perfusion and vascularization (Fig 2A). Fluid deprivation led to a significant reduction in renal artery diameter, whereas enteral resuscitation with water or ORS maintained it (-1.4 ±0.17 mm versus -0.44±0.14 mm or -0.63±0.20 mm respectively; Fig 2B; *P* < 0.02). Kidney volume significantly increased from baseline to 48 h post-burn in animals receiving water (20.0 ± 3.0 cm^3^), but did not change with fluid deprivation or animals consuming ORS groups (-0.38±5.2 and 1.2±5.5 cm^3^, respectively; *P* < 0.02; Fig 2C). Total kidney weight was similar in animals receiving water or ORS and statistically greater when compared with fluid deprivation (114.90±3.48 g or 114.20±4.50 g versus 93.58±4.26 g respectively; *P* < 0.001; Fig 2D). Lastly, the wet-to-dry ratio was lowest in the fluid-deprived group (5.01±0.27 g), and statistically higher in the water and ORS group (6.06±0.27 g and 6.06±0.38 g, respectively *P* < 0.002; Fig 2E).

### Fluid deprivation increases kidney glycogen

**Fig 3:**
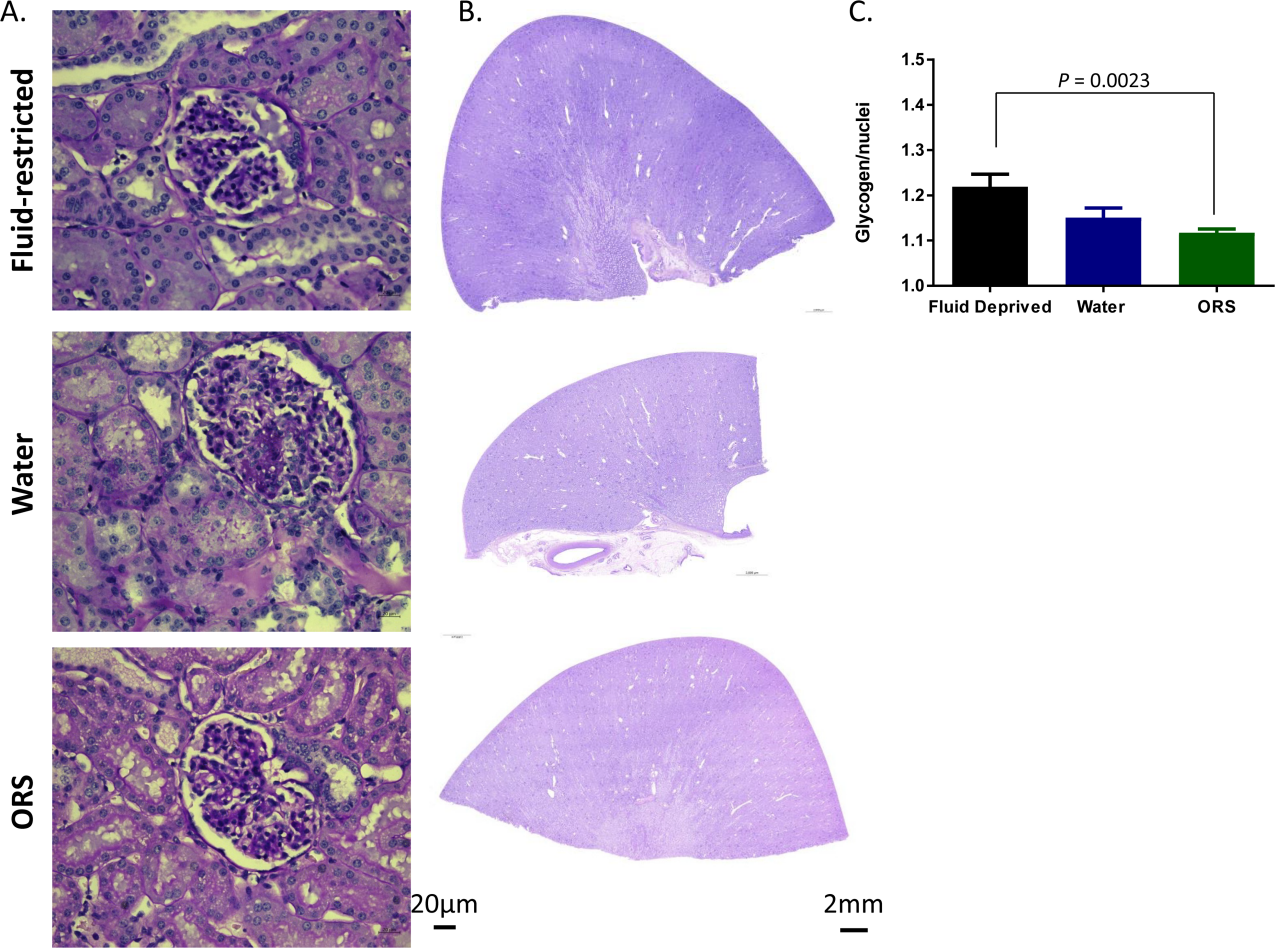
Renal glycogen content is greatest with fluid deprivation. (A) Representative H&E staining reveals hallmarks of glomerulonephririts in all groups, to include inflammatory cells (#), clotting with cell debris (*), parietal cell hypertrophy (arrow), and synechia (arrowhead) in all groups. Representation (B) and quantification of the color density reveals significantly higher glycogen content in the fluid deprived group compared with the water and ORS groups (* *P* < 0.05).

Scores for tubular degeneration were 6.2 ± 0.8, 7.2 ± 0.7, and 7.3 ± 0.3 in fluid-deprived, water, and ORS groups, respectively, and were not statistically different from each other (data not shown). Other histological findings indicate glomerulonephrirtis hallmarked by microthrombi formation, synechia and parietal cell hypertrophy (Fig 3). Moderate to severe glomereulonephritis was present in all animals in the water-deprived group, 2/6 animals in the water group, and 3/6 animals in the ORS group. Representative PAS staining reveals a darker intensity of staining in the fluid-deprived animals (Fig 3). After color deconvolution, the pink glycogen channel was normalized to the amount of nuclei (Fig 3B). As shown in Fig 3C, there is a significant increase in the glycogen content of kidneys from the fluid-deprived group when compared to the water and ORS groups (*P* = 0.012). While this may indicate a greater dependency of the kidney on gluconeogenesis as opposed to glycogenolysis, there was also greater glucosuria at 48h in the water deprived group (102.0 ± 39.8 mg/dL) than the water (39.6 ± 9.1) and ORS (22.8 ± 3.8mg/dL) groups.

### Inflammatory mediators are elevated in fluid-deprived animals

**Fig 4:**
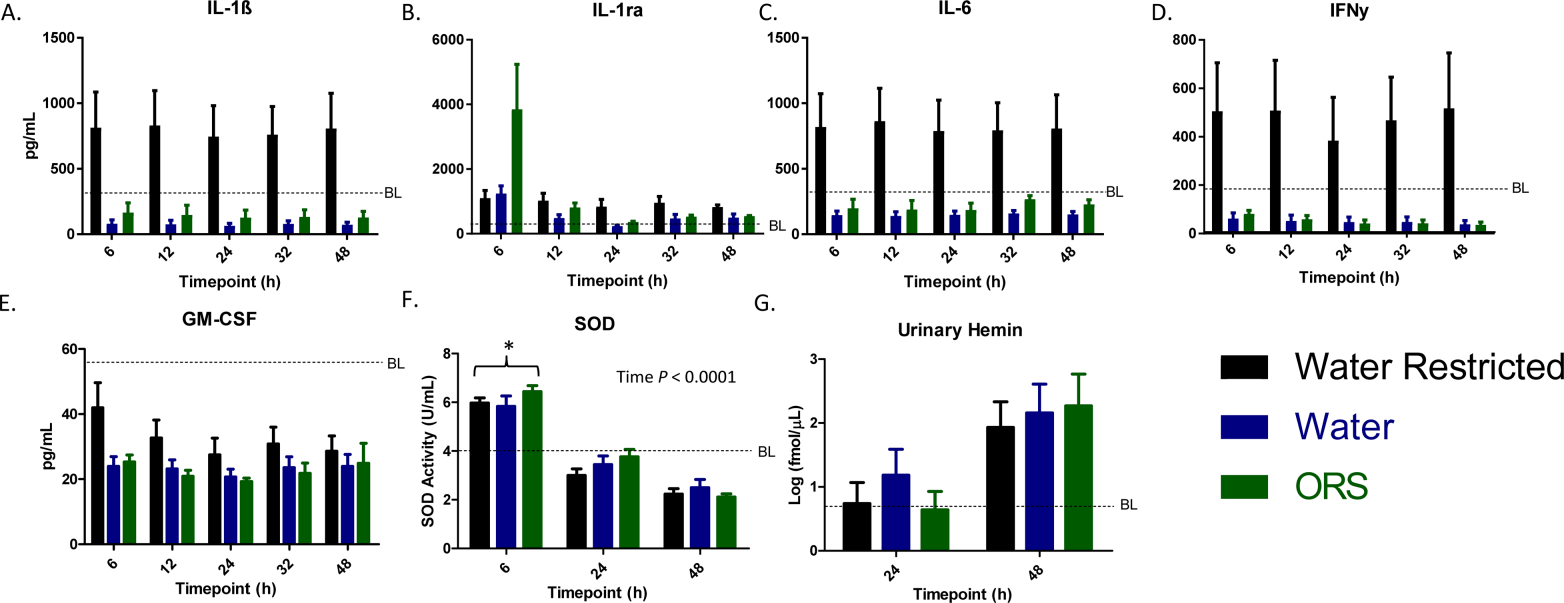
Fluid deprivation elevates circulating cytokines. Select circulating cytokines were quantified across all time points (A-E). Average of baseline values of all treatments is set as a reference line and bars represent mean ± SEM. Superoxide dismutase (SOD) activity in the plasma (F) and urinary hemin (G) was quantified at BL, 6, 24, and 48 h and presented as mean ± SEM. Asterisk denotes a significant difference (*P* < 0.05).

Pro-inflammatory and anti-inflammatory cytokine levels in plasma were quantified across time (Fig 4) and compared to averaged baseline levels. Expression of IL-1β (*P* ≤ 0.019), IL-6 (*P* ≤ 0.027), and IFNγ (*P* ≤ 0.12) was greatest in fluid-deprived animals regardless of time point; however, no time effect or differences between water and ORS were detected. Expression of IL-1ra significantly increased post-burn compared to baseline levels, and was also significantly highest in the ORS group at 6 h (*P* < 0.002). Granulocyte-macrophage colony-stimulating factor (GM-CSF) was lower than baseline levels in all animals, but was not significantly different among the groups. Circulating levels of the antioxidant enzyme SOD peaked 6 h following burn and then was significantly lower than BL by 48 h in all animals (*P* < 0.05; Fig 4F). Free hemin in the urine was elevated by an order of magnitude at 48 h in all animals (Fig 4G). However, urinary hemin was not significantly different among treatments, even when normalized to creatinine (data not shown).

## Discussion

Severe burn injury elicits massive repartitioning of intracellular and extracellular fluids leading to circulatory dysfunction (i.e., vascular leak and edema) and subsequent organ damage. These hemodynamic fluctuations are treated with large volumes of IV fluids in an attempt to maintain adequate tissue perfusion, but this therapy may be associated with various co-morbidities and exacerbate tissue edema [15, 22, 23]. Animal studies [11] and clinical investigations [10, 12, 24, 25] dating as far back as the 1940s, demonstrate the effectiveness of enteral resuscitation in the treatment for burn shock. Indeed, several reviews on oral resuscitation in mass casualty care have suggested that provision of an enteral salt solution is effective for the treatment of burns [13, 26]. Despite this, there is a lack of evidence on its efficacy in ameliorating organ dysfunction after burn injury. The primary findings of this study are that AKI occurs in the acute time frame post-burn and enteral fluids (in the absence of IV resuscitation) have protective effects on burn-induced renal damage/dysfunction, with the addition of salts conferring extra benefit.

Results presented herein compare two oral fluids and a fluid-deprived group after a 40% TBSA surface contact burn in swine. Following burn injury all animals presented with elevated clinical parameters for inflammation and AKI by 6 h. The efficacy of enteral resuscitation with ORS was most convincingly demonstrated with the improvement in GFR. Kidney recovery was also monitored via CT and showed oral fluids maintained volume and renal artery diameter. In the case of perfusion (i.e., Hounsfield units), no significant differences were detected among fluid deprived and enterally resuscitated swine (data not shown), perhaps due to reduced kidney weight. In this regard, these (Hounsfield) units may be a poor indicator of renal perfusion. Greater post-mortem kidney weights and wet-dry ratios in animals receiving fluids further demonstrated that enteral resuscitation maintained kidney perfusion. This could also be interpreted as development edema; however, significant edema was not seen with histology. Additionally, the former is further supported by a significant inverse relationship between plasma creatinine and kidney weight (*P* = 0.04; r^2^ = 0.17) (data not shown), thus strengthening the relationship between perfusion and function.

While the ORS used in this study was selected based on its track record of use, there are several different rehydration solutions commercially available. Solution formulations vary, yet the basic composition consists of an isotonic or hypertonic sodium, chloride, and carbohydrate solution. The therapeutic efficacy of oral resuscitation with sodium lactate was demonstrated by Fox [10] who provided a preliminary detailed report on nine patients who improved with treatment. A more recent study showed a oral rehydration therapy containing rice-based (Ceralyte®) carbohydrates reduced IV fluid requirements in burn patients [27]. While a clinical reduction in IV fluid requirements may circumvent the associated edema and co-morbidities, enteral fluids alone may be insufficient to ensure adequate end-organ perfusion. Still, as IV fluid requirements are driven off of urine output, the current report also indicates a reduction in IV fluids may be possible by administering ORS. Of note, target urine output is 0.5–1.0 mL/kg/h and our groups span this range with ORS achieving urine output of 1.07±0.21 mL/kg/h and fluid restricted at 0.52±0.03 mL/kg/h.

The efficacy of ORS stems from the sodium-dependent absorption of glucose monomers in villous cells of the small intestine via the sodium-dependent glucose co-transporter (SGLT1) [28, 29]. Specifically, transport across SGLT1 requires Na+/K+ ATPase pump to create a downward sodium gradient for the movement of sodium ions and glucose across the apical membrane. While plasma glucose was temporally affected after burn injury (Table 1), this was not affected by the glucose present in ORS and could be indicative of feeding schedule. However, glucose present in the urine post-burn may corroborate this finding as a stress response to the injury. The ensuing osmotic gradient creates a hypertonic environment in the paracellular space [30], essentially replacing plasma volume [15]. The importance of solutes in these solutions is demonstrated in the current study by the failure of water to increase urine output and GFR. Still, further work is needed to optimize oral rehydration solutions. Solutions containing glucose polymers (e.g., rice-based carbohydrates mentioned above),short-chain fatty acids may potentiate absorption through the colon and the small intestine [28]. The effects of oral resuscitation fluid with pyruvate on the intestines have recently been investigated in 35% TBSA scalded rats. Intestinal absorption of sodium and water was increased with a pyruvate-supplemented ORS, although water alone was not tested [31]. Further, if large volumes of oral resuscitation conserve the gut mucosal lining, it would be noteworthy as disruption grants access of bacteria into circulation [13, 32]. For this study, animals did not develop bacteremia (even in the water deprived group). Whether this is because of maintained enterocyte integrity or the ability of the porcine immune system to sufficiently neutralize bacteria remains to be elucidated.

One of the primary tools clinicians use to drive the volume of IV fluids given is urine output, due to its estimation of end-organ (kidney) function. In this study ORS’s superiority to water in increasing urine output and GFR would likely result in a decrease in the amount of IV fluids administered and potentially, edema. As indicated in Fig 1 the volume of fluid ingested, although slightly lower, was not different in animals given ORS, although they had nearly triple the urine output within the span of the study. Kramer et al. [13] reported a Chinese publication in a 30% TBSA burned canine model given orally a volume of a glucose, NaCl, and NaHCO_3_ mix at an osmolality that is similar to ORS (347 vs. 331, respectively) or a hypotonic version according the Parkland formula (4 mL/kg/TBSA). Similar to our data, improvements in urine volume excreted were noted with animals receiving the hypertonic solution. Moreover, a recent study randomized patients up to 20% TBSA to receive either enteral or IV fluids found that the only significant difference was higher urine output in the patients receiving enteral fluids. While the optimal type of enteral fluid for use in burns is unknown, the current study advocated for their use in improving creatinine clearance.

Similarly, the efficacy limit of enteral fluids in terms of volume also remains to be answered. The calculated volumes used in the current study (1.75 ml/kg/%TBSA) are slightly lower but comparable to IV guidelines given by the modified Brooke formula (2 ml/kg/%TBSA), and much less than the Parkland formula (4 ml/kg/%TBSA). The operational difference when examining the availability in austere environments lies in the fact that several liters of sterile IV fluids are heavy, while ORS is available in lightweight sachets that can be reconstituted with any potable water. Moreover, in disaster scenarios no special training is required to administer oral fluids. While these resource-limited environments were our initial motivation for examining enteral (but not IV) fluids, this potential treatment strategy has received strong interest due to the recent nation-wide shortage of IV fluids after the hurricanes in Puerto Rico. The questions of how much enteral fluid is effective, how long it might be effective, and in what injury severity it will be effective, remain unanswered. The volume of ORS used in this study is far below the absorptive capacities of the intestine, which is reported to be around 20 L/day [33]. This amount is approximately equal to Parkland estimations for a 70-kg individual sustaining a 70% TBSA injury. Certainly in this case, IV resuscitation would be necessary, although total IV requirements could potentially be reduced with administration of enteral fluids.

In this regard, while large volumes of IV crystalloid is the standard of care, it has been shown that too much crystalloid is detrimental in burn patients (i.e., fluid creep) [34]. For example, large volumes of crystalloids can result in acute respiratory distress syndrome, compartment syndromes and MOD [35–37]. Crystalloid use was promulgated in the surgical literature for decades with studies done supporting mortality benefits for ‘supranormal’ hemodynamic parameters [38–40]. However, crystalloid is becoming increasingly recognized as a detrimental fluid because it is acidic, pro-inflammatory, and results in hemodilution. As such, in other forms of trauma (e.g., hemorrhagic shock) there has been a recent movement away from massive crystalloid resuscitation to other alternatives, or more moderate volumes of crystalloids. It would be interesting to see if IV fluid administration produced pro- or anti-inflammatory effects in this model, as enteral fluids (both water and ORS) reduced cytokine levels fairly non-specifically. However, ORS was able to increase the anti-inflammatory IL1ra and the antioxidant SOD when compared to the other groups at 6 hours. While this indicates that enteral fluids may buy extra time during triage or transport of patients, the effects of IV fluids on inflammation in this model remains the province of future investigation.

While this transition away from crystalloids in hemorrhagic shock patients took decades, it was logical given that blood was being lost, and should be replaced by whole blood [41–43]. While burn patients also have massive fluid losses, there is no direct blood loss per se, which is the justification for crystalloid infusion. Aside from transepidermal evaporation, there is massive fluid and protein shifts out of the intravascular space into visceral organs, which may explain a trend toward resuscitation with plasma [44]. To combat this, the contents of enteral fluid may be formulated to drastically alter the absorptive capacity of the small intestine. For example, one group has utilized ORS containing different compositions of amino acids, and shown that this affects ileal absorption of carbohydrates, amino acids, and electrolytes in radiation injury [45, 46]. Taken together, much more research needs to be done in order to realize a closer approximation to the fluid lost in the thermally injured population.

Limitations of this study include the acute nature of these experiments, with no indication on the long term effects of ORS. For example, we observed no critical imbalances in plasma electrolytes in animals receiving ORS, but cannot predict the effects of long-term administration of ORS (S3 Fig). Kidney tissue was harvested and processed at only one timepoint (48h) to evaluate the acute resuscitation period. Another limitation is that IV fluid administration which is the current standard of care (albeit with substantial variation in resuscitation protocols) was not included as a treatment group. While it is hard to imagine inferiority of IV fluids to enteral fluids, it also may be difficult to prove the opposite in this model, as enteral fluids alone sufficiently supported urine output and returned creatinine values to baseline. Regardless, future investigations into the efficacy of different types and volumes of IV fluids are needed. Although the severity of injury and demographics/ compliance of our subjects were uniform, future research could also explore volumes and types of both IV and enteral routes of administration to identify which patients may benefit from either route. Finally, it is highly unlikely for a burn victim to be deprived IV fluids and other definitive clinical care except potentially under austere military or wilderness environments or civilian mass casualty situations, where supplies may be extremely limited.

## Conclusions

Data presented here suggest that enteral resuscitation is efficacious with regard to burn-induced renal dysfunction, and that inclusion of salts (i.e., ORS) provides additional benefit. These results will help inform the design of clinical studies in humans to assess its safety and efficacy. Not only could this save lives in prolonged field care, mass casualty, or wilderness medicine scenarios, but its incorporation in definitive clinical care may reduce overall IV fluid requirements and the ensuing complications of resuscitation of the extravascular space. In patients receiving nasogastric tubes, this strategy could easily be incorporated into routine care. Future research should explore the enteral route for resuscitation and compare outcomes to the current standard of care.

## Supporting information

S1 Fig ARRIVE Guidelines. (PDF)

S2 Fig Veterinary support branch health and behavior check form. Veterinary technicians are required to monitor animal health and behavior daily of all animals (i.e. healthy and injured) on site. Technicians are trained to spend time with each individual animal to inspect and monitor well-being. This health and behavior check is independent of animal monitoring performed by research group. If abnormal animal behavior is recorded by trained staff, the head technician and/or veterinarian is notified for additional care.
(PDF)

S3 Fig Swine pain and distress assessment score sheet. Parameters according to swine behavior and activity are monitored and reported following injury. Research team completes assessment and records results, if unsatisfactory behavior or severe complications are seen on call veterinarians are consulted for additional care.
(PDF)

S4 Fig Plasma electrolyte levels following burn injury. (A) Sodium, (B) Potassium, and (C) Chloride levels in plasma following burn injury at 0, 6, 12, 24, 32, and 48 h in fluid deprived, water, and ORS treated swine. Means ± SEM with a different superscript letter are significantly different (*P* < 0.05) between treatments for indicated time point and a ᵻ indicates a significant (*P* < 0.05) difference from the BL value.
(TIF)
