# Supplementary material for: Enteral resuscitation with oral rehydration solution to reduce acute kidney injury in burn victims: Evidence from a porcine model
Source: PLoS One. 2018 May 2;13(5):e0195615. doi: 10.1371/journal.pone.0195615 (PMC5931460; doi:10.1371/journal.pone.0195615)
Supplement: S1 Fig — (PDF) [file pone.0195615.s001.pdf]

The ARRIVE Guidelines Checklist

	Item	Recommendation
Title	1	Enteral Resuscitation with Oral Rehydration Solution to Reduce Acute Kidney Injury in Burn Victims: Evidence from a Porcine Model
Abstract	2	Intravenous (IV) resuscitation of burn patients has greatly improved outcomes and become a cornerstone of modern burn care. However, the heavy fluids and vascular access required may not be feasible in austere environments, mass casualty, or delayed transport scenarios. Enteral resuscitation has been proposed for these situations; we sought to examine the effectiveness of this strategy on improving burn-induced kidney injury. Anesthetized Yorkshire swine sustaining 40% TBSA full-thickness contact burns were randomized to three groups (n=6/group): fluid deprivation, ad libitum water access, or 70 mL/kg/d Oral Rehydration Salt solution (ORS). Urine and blood were collected at baseline (BL), 6, 12, 24, 32, and 48h post-burn, at which point tissue was harvested and CT angiography performed. Although fluid consumption by ad libitum and ORS groups were matched (132±54mL/kg versus 120±24mL/kg, respectively), ORS intake increased urine output compared with water and no water (1.07±0.21 mL/kg/h versus 0.68±0.31 mL/kg/h, and 0.52±0.03 mL/k/h respectively). Plasma creatinine peaked 6h following burn (1.67±0.07mg/dL) in all animals, but at 48h was comparable to BL in animals receiving water (1.23±0.06mg/dL) and ORS (1.30±0.09mg/dL), but not fluid deprived animals (1.56±0.05mg/dL) (P<0.05). Circulating levels of blood urea nitrogen steadily increased, but also decreased by 48h in animals receiving enteral fluids (P<0.05). Water deprivation reduced renal artery diameter (-1.4±0.17mm), whereas resuscitation with water (-0.44±0.14 mm) or ORS maintained it (-0.63±0.20 mm;P< 0.02). Circulating cytokines IL-1β, IL-6, IFNγ, and GM-CSF were moderately elevated in the fluid-deprived group. Taken together, the data suggest that enteral resuscitation with ORS rescues kidney function following burn injury. Incorporating enteral fluids may improve outcomes in resource-poor environments and possibly reduce IV fluid requirements to prevent co-morbidities associated with over-resuscitation. Studies into different volumes/types of enteral fluids are warranted. While ORS has saved many lives in cholera-associated dehydration, it should be investigated further for use in burn patients.
Introduction		

Background	3	While IV fluid resuscitation remains the standard treatment for burn patients, the efficacy of oral rehydration therapy has been proposed for decades (10-12). Past clinical trials and animal experiments utilized various formulations of simple electrolyte solutions and found them effective for the treatment of burn injury (13). In disaster or resource-limited situations, enteral fluids may be the only option due to a lack of IV fluids or an inability to gain vascular access.<br>The Oral Rehydration Salt solution (ORS) according to the World Health Organization (WHO) is a simple formula that contains glucose, sodium chloride, potassium chloride, and trisodium citrate. ORS has been successfully used for decades to save millions of lives in third world countries from dehydration secondary to severe diarrhea in conditions such as cholera (14). This suggests the feasibility of rapidly mobilizing these simple treatments in the wake of large-scale mass casualty incidents.
Objectives	4	The objective of this study was to identify if ORS is effective at improving burn-induced kidney injury in austere environments of during delayed standard care scenarios.
Methods		
Ethical Statement	5	This study has been conducted in compliance with the Animal Welfare Act, the implementing Animal Welfare Regulations, and the principles of the Guide for the Care and Use of Laboratory Animals.
Study Design	6	Anesthetized Yorkshire swine sustaining 40% TBSA full-thickness contact burns were randomly allocated to one of three treatments following thermal injury: fluid deprived (n=6), ad libitum water access (n=6), or 70 mL/kg/d of ORS (ORS; n=6) for 48h.
Experimental Procedures	7	Thermal Injury section in Materials and methods
Experimental Animals	8	Eighteen pre-pubertal female Yorkshire swine weighing 40.2±2.1 kg.
Housing and Husbandry	9	Animals recovered and were kept in a metabolic cage (dimensions 41'L x 16' W x 44' H).
Samples Size	10	18
Allocating animals to experimental groups	11	6 animals chosen randomly to each group.
Experimental outcomes	12	Please refer to Results section.
Statistical methods	13	Please refer to Statistical analysis section under Materials and methods.
Results		
Baseline data	14	Please refer to Results section.
Numbers Analyzed	15	18
Outcomes and Estimation	16	Please refer to Results section.
Adverse Events	17	none
Discussion		Please refer to Discussion section.

Interpretation/Scientific Implications	18	Please refer to Discussion section.
Generalisability/ Translation	19	Please refer to Conclusion section.
Funding	20	Funded by US Army Medical Research Materiel Command W81XWH-16-2-0041.
