# Supplementary material for: Enteral resuscitation with oral rehydration solution to reduce acute kidney injury in burn victims: Evidence from a porcine model
Source: PLoS One. 2018 May 2;13(5):e0195615. doi: 10.1371/journal.pone.0195615 (PMC5931460; doi:10.1371/journal.pone.0195615)
Supplement: S2 Fig — Veterinary technicians are required to monitor animal health and behavior daily of all animals (i.e. healthy and injured) on site. Technicians are trained to spend time with each individual animal to inspect and monitor well-being. This health and behavior check is independent of animal monitoring performed by research group. If abnormal animal behavior is recorded by trained staff, the head technician and/or veterinarian is notified for additional care. (PDF) [file pone.0195615.s002.pdf]

USAISR Veterinary Support Branch
Health and Behavior Check Form

Species		Room #			Date, temp, hum%, BAR, and Technician name are mandatory												
					**Food, Water/Gel**: Check if missing **Total** = total number of animals in the room **Flag**: Check if activated												
**Month:**		**Year:**		**Techs:**	**Primary:**				**Alternate:**					**Weekend:**			
**Day**	**Date**	**Temp**	**Hum%**	**Total**	**BAR**	**Flag**	**Eyes**	**Resp**	**Feet**	**Coat**	**Stool**	**Food**	**Water**	**Gel**	**EE**	**Initials**	**VCO**
**Mon**																	
**Tue**																	
**Wed**																	
**Thu**																	
**Fri**																	
**Sat**																	
**Sun**																	

EYES	Resp	Coat	Feet	Stool	**Standard enrichment (EE)** for rodents include rodent foraging material, cage furniture (hut), and a nylabone.
W=Watery	ND=Nasal Discharge	D=Dirty	NWB=Non-Weight Bearing	S=Soft	
D=Discharge	W=Wheezing	O=Other		W=Watery	
O=Other	P=Panting	S=Scratches			

Species	Normal Behaviors	Abnormal/Maladaptive Behaviors
**Mice**	Nocturnal, grooming, nest building, gnawing, thigmotaxis(remains close to walls), foraging	Barbering, fighting (especially in males), food grinding stereotypy
**Rats**	Nocturnal, nest building and burrowing, thigmotaxis foraging, coprophagic, gnawing, squeak, grunt	Barbering, stereotypy, abnormal hyperactivity, tail vibration
**Rabbits**	Non-aggressive, exploratory, "frisky hopping", chin rubbing lay flat on the ground, spraying, coprophagic	Barber or hair pulling, cage nosing or mouthing, digging, thumping, tooth chattering or grinding, honking, screaming
**Pigs**	Rooting, foraging, nesting behavior at night, wallowing	Vocalization, frantic gasping, excitability, escape behavior aggression, teeth grinding, foaming saliva, biting, slashing
**Sheep**	Rooting, foraging, kicking, pawing, rumination	Wool sucking, stereotypy, chewing, nosing pen, charging
**Goats**	Foraging, rumination	Aggression, charging, head butting, biting, stereotypy

**Comments** (*Record any abormal animal behavior or facility issues in the comments section*)	
**Mon**	
**Tue**	
**Wed**	
**Thu**	
**Fri**	
**Sat**	
**Sun**	
