# Supplementary material for: Enteral resuscitation with oral rehydration solution to reduce acute kidney injury in burn victims: Evidence from a porcine model
Source: PLoS One. 2018 May 2;13(5):e0195615. doi: 10.1371/journal.pone.0195615 (PMC5931460; doi:10.1371/journal.pone.0195615)
Supplement: S3 Fig — Parameters according to swine behavior and activity are monitored and reported following injury. Research team completes assessment and records results, if unsatisfactory behavior or severe complications are seen on call veterinarians are consulted for additional care. (PDF) [file pone.0195615.s003.pdf]

APPENDIX F

SWINE PAIN/DISTRESS ASSESSMENT SCORE SHEET								
Parameter	**Animal ID:_____________**<br>**Protocol #_____________ PI:_____________**	Score	Date/ Time/ Initial	Date/ Time/ Initial	Date/ Time/ Initial	Date/ Time/ Initial	Date/ Time/ Initial	Date/ Time/ Initial
**General Appearance**	Normal (eyes/nose clear, relaxed posture)<br>Abnormal Posture (hunched back, leaning for support, shifting weight), increased respiratory rate, skin discoloration (blotchy)<br>Fixed gaze, persistent twitching or trembling, excessive foaming at mouth, hyperventilation w/mouth breathing, head pressing	0<br>2<br>3						
**Natural Behavior**	Normal (playing, foraging, rooting, standing, social interaction with observer)<br>Less active, lethargic/depressed, decreased social interaction with observer (isolated at back of cage), increased aggression<br>Inactive, recumbent, increased vocalization/squealing – esp when approached/ touched by observer	0<br>2<br>3						
**Fecal/Urinary Output**	Normal (multiple piles of feces, urine present)<br>Decreased (≤ 1 pile feces, only small amount of urine)<br>Absent or severely decreased, watery/bloody diarrhea	0<br>1<br>3						
***Body Weight/ **Body Condition Score (BCS)**<br>Initial _______kg	Normal (no / mild weight loss), BCS of 3<br>3-9% total wt loss, BCS 2<br>≥10% wt loss in a 24 hour period or ≥15% total wt loss, BCS 1	0<br>1<br>3	____kg	____kg	____kg	____kg	____kg	____kg
**Appetite**	Normal appetite, eats all enrichment<br>Reduced appetite, eats some enrichment<br>No appetite, not eating enrichment	0<br>1<br>3						
**Wound Assessment**	None/healed<br>Mild redness, +/- swelling<br>Moderate redness, +/- swelling<br>Discharge, necrosis, dehiscence (possible septicemia)	0<br>1<br>2<br>3						
**Mobility/Lameness**<br>(any animal unable to stand and ambulate (with assistance) for = or > 24 hrs will be promptly evaluated for euthanasia)	Normal ambulation<br>Slight lameness in one/both legs, short periods of standing/walking (unassisted)<br>Lame, limited ability to stand and walk (assisted)<br>Recumbent – little response when prodded	0<br>1<br>2<br>3						

INSTRUCTIONS FOR USE: Scoring is to be performed and recorded for a minimum of 3 days post-operatively and will continue as indicated below. The following score key will be used. The highest score in any of the above categories will dictate the level of intervention and number of observations. Interventions will be annotated on the scoring sheet in the comments section.

0 – Routine Observations (no intervention scoring) **1** – Intervention scoring 1x per day
2 – Intervention scoring recommended 2x per day (unless exempted by vet); implement appropriate interventions (i.e.SC fluids, increased dietary enrichment, thermal support, re-apply skin staples/sutures)
3 – Consult veterinarian and/or PI/AI to discuss further intervention (analgesia, antibiotics, incision closure, euthanasia, etc.)

**Other than initial body weight, daily body weights are optional and not required unless handling of the animal is necessary for experimental reasons.*
******BCS 3 – Ideal*** *– hipbones and spinous processes can be felt with firm pressure*
BCS 2 – Thin *– hip bones and spinous processes are easily felt without firm pressure*
BCS 1 – Very Thin *– hip bones and spinous processes are very prominent with no fat cover, pig is visibly thin*

Analgesia administered:
Buprenorphine SR LAB Dose: ____________ Route: ______ Time: ________Initials: _______ (Other) ___________________Dose: ____________ Route: ______ Time: _______ Initials: _______
Dose: ___________ Route: ______ Time: _______ Initials: ________
Comments:
____
____
____
