# Supplementary figures and images for: Enteral resuscitation with oral rehydration solution to reduce acute kidney injury in burn victims: Evidence from a porcine model
Source: PLoS One. 2018 May 2;13(5):e0195615. doi: 10.1371/journal.pone.0195615 (PMC5931460; doi:10.1371/journal.pone.0195615)

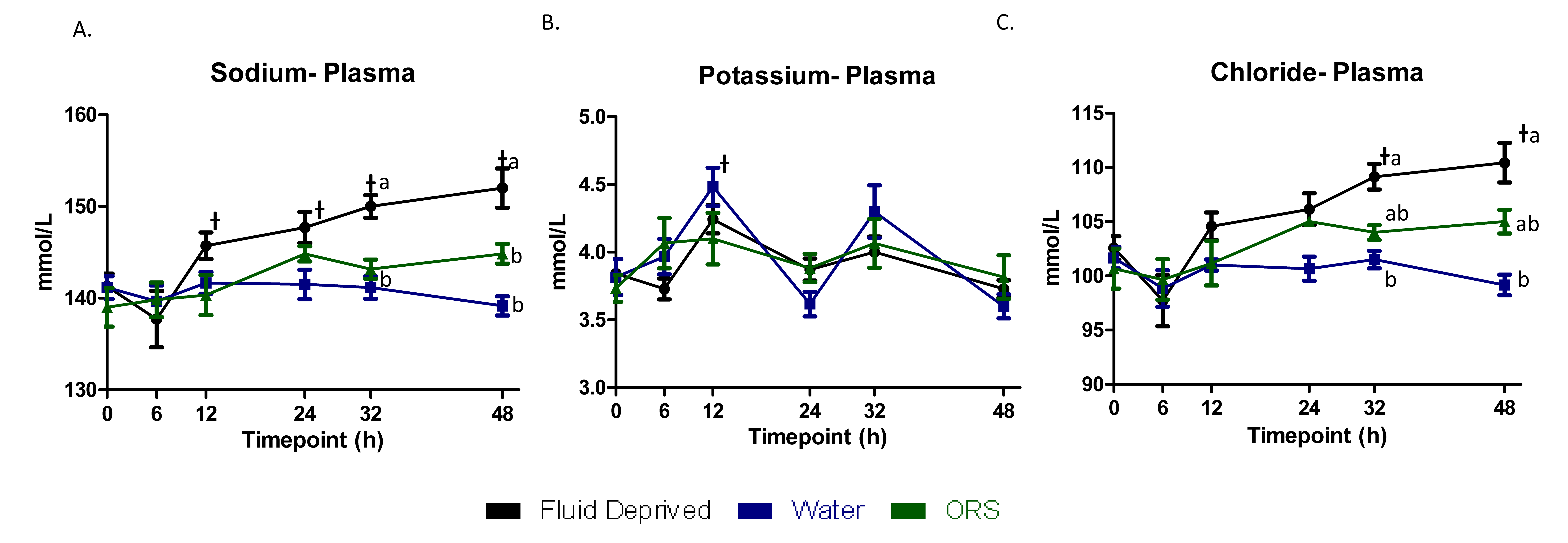

Supplement: S4 Fig — (A) Sodium, (B) Potassium, and (C) Chloride levels in plasma following burn injury at 0, 6, 12, 24, 32, and 48 h in fluid deprived, water, and ORS treated swine. Means ± SEM with a different superscript letter are significantly different (P < 0.05) between treatments for indicated time point and a ᵻ indicates a significant (P < 0.05) difference from the BL value. (TIF) [file pone.0195615.s004.tif]
